# Supplementary material for: Association between oral microbiome and seven types of cancers in East Asian population: a two-sample Mendelian randomization analysis
Source: Front Mol Biosci. 2023 Nov 21;10:1327893. doi: 10.3389/fmolb.2023.1327893 (PMC10702768; doi:10.3389/fmolb.2023.1327893)
Supplement: Supplementary file 1 [file DataSheet1.ZIP › Supplementary Materials/MR plots of saliva/saliva═╝/Pancreatic cancer/pheno.3596_to_pancreatic cancer_forest.pdf]

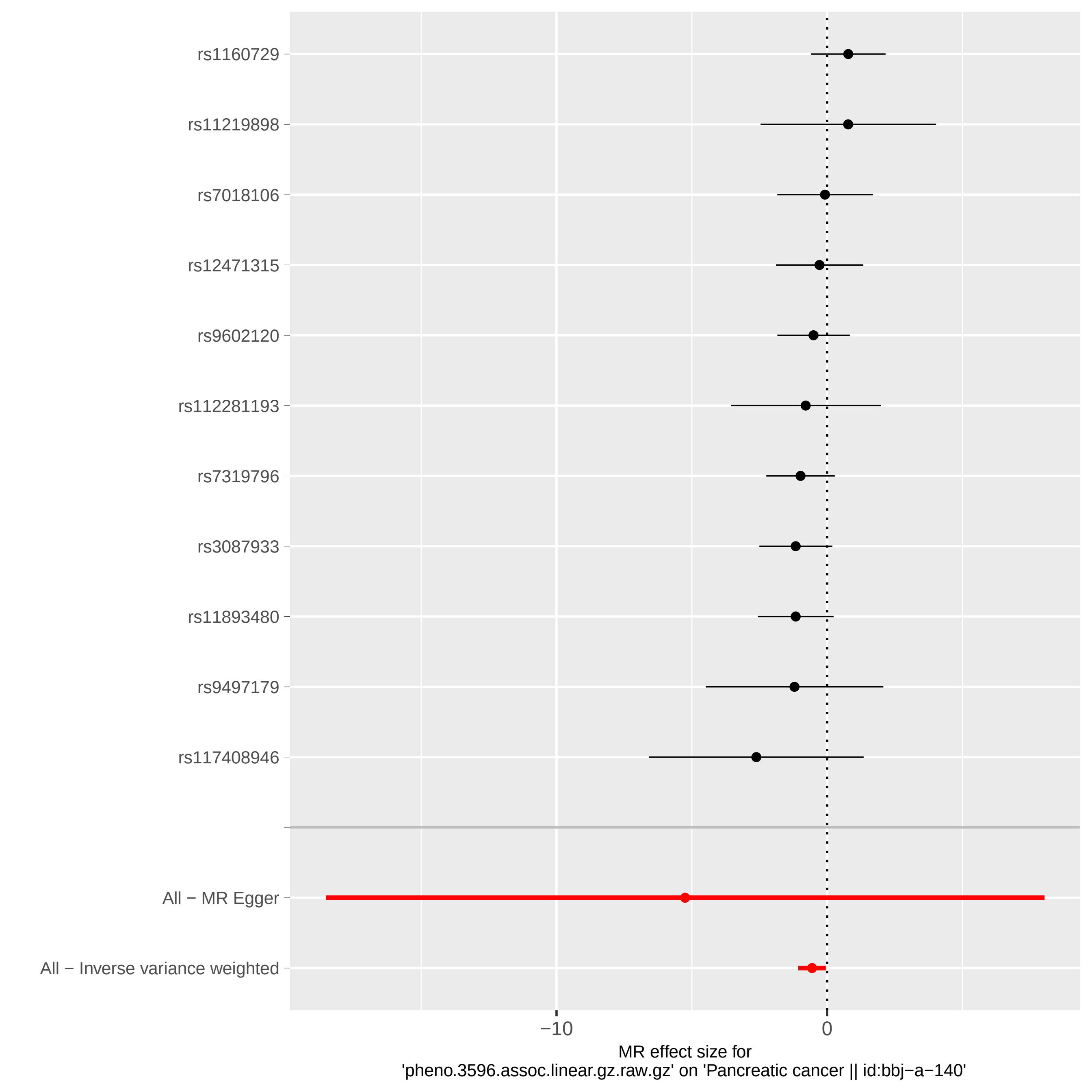

rs1160729
rs11219898
rs7018106
rs12471315
rs9602120
rs112281193
rs7319796
rs3087933
rs11893480
rs9497179
rs117408946
All − MR Egger
All − Inverse variance weighted
−10
0
MR effect size for
'pheno.3596.assoc.linear.gz.raw.gz' on 'Pancreatic cancer || id:bbj−a−140'
